# T lymphocyte insensitivity to corticosteroids in chronic obstructive pulmonary disease

**DOI:** 10.1186/1465-9921-13-20

**Published:** 2012-03-14

**Authors:** Manminder Kaur, Lucy JC Smyth, Paul Cadden, Seamus Grundy, David Ray, Jonathan Plumb, Dave Singh

**Affiliations:** 1 University of Manchester, Manchester Academic Health Science Centre, NIHR Translational Research Facility, University Hospital of South Manchester Foundation Trust, Southmoor Road, Manchester, UK M23 9LT; 2 School of Medicine, University of Manchester, Oxford Road, Manchester, UK M13 9PT; 3 Centre for Parasitology and Disease, School of Environment and Life Sciences, University of Salford, Salford, UK M5 4WT

**Keywords:** COPD, Lymphocytes, Corticosteriods

## Abstract

**Background:**

There are increased numbers of activated lymphocytes in the lungs of chronic obstructive pulmonary disease (COPD) patients. The clinical benefits of corticosteroids in COPD patients are limited. Our hypothesis is that lymphocytes play a role in this corticosteroid insensitivity.

**Objectives:**

To investigate the effects of the corticosteroid dexamethasone on lung lymphocyte cytokine production from patients with COPD compared to controls.

**Methods:**

Cultured airway lymphocytes obtained by bronchoscopy from healthy non-smokers (HNS), smokers (S) and COPD patients were stimulated with phytohaemagglutinin (PHA) & phorbol myristate acetate (PMA), +/- dexamethasone. Supernatants were assayed for interleukin (IL)-2 and interferon (IFN)γ. Immunofluoresence was used to analyse changes in CD8 glucocorticoid receptor (GRα and GRβ) expression.

**Results:**

The inhibition of PHA/PMA stimulated IFNγ production by dexamethasone was reduced in COPD patients compared to HNS (*p *< 0.05 at concentrations from 0.1-1 μM). There was also a significant reduction (*p *< 0.05) in the mean inhibitory effect at 1 μM in COPD patients (54.1%) compared to smokers (72.1%), and in smokers compared to HNS (85.5%). There was a numerically reduced effect of dexamethasone on IL-2 production that did not reach statistical significance. There was no difference in GRα and GRβ expression in follicular CD8 cells between COPD patients (50.9% and 30.4% respectively) and smokers (52.9% and 29.7% respectively).

**Conclusions:**

IFNγ production from COPD airway lymphocytes is corticosteroid insensitive. This phenomenon may be important in the poor clinical response often observed with corticosteroids.

## Background

Chronic obstructive pulmonary disease (COPD) is a progressive inflammatory airway disease. There are increased numbers of lymphocytes, particularly CD8 cells, in the lungs of COPD patients leading to the suggestion that COPD has an autoimmune component [1-3]. CD8 cells are increased in the airway lumen, sub-epithelium and parenchyma and their numbers increase proportionate to disease severity [1]. T lymphocytes from the airways of COPD patients produce pro-inflammatory cytokines, with evidence of both Th1 and Th2 cytokine production [4-8]. Furthermore, there is an increase in organised lymphoid structures called follicles within the lung parenchyma and associated with the bronchial tree [9]. These follicles may function as antigen presenting sites, further supporting the hypothesis that COPD has an autoimmune component [10].

Corticosteroids are the most widely used anti-inflammatory drug in COPD patients. Corticosteroids bind to the cytoplasmic glucocorticoid receptor (GR), forming a complex that can suppress inflammatory gene transcription [11]. However, the clinical benefits of corticosteroid therapy in COPD are limited [12-14]. Using peripheral blood derived lymphocytes, it has been demonstrated that corticosteroids have reduced effects on phytohaemagglutinin (PHA)-induced lymphoproliferative responses in patients with rheumatoid arthritis, ulcerative colitis and corticosteroid resistant asthma [15-18]. Our hypothesis was that COPD airway T lymphocytes also have reduced sensitivity to the effects of corticosteroids.

We report an investigation of the corticosteroid sensitivity of airway T lymphocytes from COPD patients compared to controls. The effect of dexamethasone on bronchoalveolar lavage (BAL) T lymphocyte cytokine production was investigated. In order to establish whether altered GR expression plays a role in corticosteroid sensitivity in lymphocytes; GR expression was quantified in CD8 cells of COPD patients and smoking controls.

## Methods

### Study subjects

14 COPD patients, 10 smokers with normal lung function and 10 healthy non smokers (HNS) were recruited (demographics shown in Table 1). Some of these patients were recruited specifically to undergo a bronchoscopy for research purposes (6 COPD patients, 3 smokers and 7 HNS). The remaining patients were undergoing clinical investigational bronchoscopies for the following reasons; haemoptysis (3 COPD patients, 1 smoker, 3 HNS), unexplained shortness of breath or weight loss (2 COPD patients, 2 smokers), and investigation of abnormal chest X-ray findings suggestive of lung cancer (3 COPD patients and 4 smokers). Subjects with any other pulmonary conditions including history of asthma were excluded. For immunofluorescent analysis, 10 COPD patients and 11 smokers with normal lung function, undergoing surgical resection for suspected or confirmed lung cancer were recruited. Tissue blocks were preselected for this study based on the presence of at least one inflammatory follicle. COPD patients were diagnosed based on a history of smoking (> 10 pack years), typical symptoms and airflow obstruction (FEV_1 _< 80% predicted, and FEV_1_/FVC ratio < 0.7) [19]. All subjects gave written informed consent. The study was approved by the local research ethics committee.

**Table 1:** Subject Demographics data are expressed as means (SD)

	COPD N = 14	Smokers N = 10	HNS N = 10	COPD N = 10 IF	Smokers N = 11 IF
Sex (F/M)	4/10	3/7	5/5	4/6	5/6

Age (years)	66.9 (7.3)	62.4 (20.6)	39.5 (22.4)	65.2 (6.8)	66.1 (8.6)

Gold Stage (1/2/3)	0/13/1	N/A	N/A	3/6/1	N/A

FEV_1 _(L)	1.8 (0.6)	2.0 (0.9)	3.4 (1.2)	2.01 (0.5)	2.06 (0.5)

FEV_1_% predicted	62 (12)	98 (19)	100 (15)	70.4 (11.5)	86.4 (19.3)

FEV_1_/FVC ratio	56 (13.8)	77 (5.7)	83 (6.0)	60.8 (11.1)	73.8 (7.7)

Pack year history	40.7 (26.4)	37.2 (28.5)	0	49.2 (39.7)	62.3 (29.5)

Current/Ex smoker	5/9	5/5	N/A	9/1	11/0

ICS Users	6	0	0	2	0

BAL Yield (mls)	91.0 (76.3)	115 (109)	190 (131)	N/A	N/A

Total Cell Yield/ml (× 10^6^)	0.23	0.28	0.13	N/A	N/A

Total Lymphocytes (%)	6.9	6.6	7.6	N/A	N/A

IF = immunofluoresence

### Cell collection

BAL was collected from the upper lobes, or a lobe not affected by radiographic or endobronchial abnormalities: The bronchoscope was wedged in the bronchus and a maximum of 4 × 60 ml aliquots of pre-warmed sterile 0.9% NaCl solution were instilled into each lobe. The aspirated fluid was stored on ice before filtration (100 μm filter, Becton Dickenson). The filtrate was centrifuged (400 *g*/10 min at 4°C) and the cell pellet resuspended in RPMI 1640 medium supplemented with 2 mM L-glutamine, 100 U/ml penicillin, and 100 μg/ml streptomycin. Viable cell counts were determined by trypan blue exclusion (Neubauer hemocytometer) and adjusted to 1 × 10^6 ^cells/ml. Macrophages were depleted by adherence by 90 minute incubation in a 24 well plate at 37°C, 5% CO_2_. The non adherent cell count was adjusted to 2 × 10^6 ^cells/ml in supplemented RPMI 1640 medium with additional 10% (vol/vol) fetal calf serum. This cell suspension was then used for cell culture.

### Cell Culture

1 × 10^5 ^cells (non adherent BAL), were seeded in a 96 well plate +/- dexamethasone (1, 0.1 & 0.01 μM) for 2 hours at 37°C, 5% CO_2 _in 200 μl RPMI-1640 media supplemented with 2 mM L-glutamine, 100 U/ml penicillin, and 100 μg/ml streptomycin and 10% FCS (v/v). Subsequently lymphocytes were activated by addition of PHA and phorbol myristate acetate (PMA), 10 μg/ml each. Cells were further incubated for 24 hours. This time point was chosen based on initial time course experiments over 2 days in 3 smoking subjects (data not shown). Supernatants (180 μl) were harvested by plate centrifugation 10 mins, 400 g, 4°C and transferred to a fresh 96 well plate for storage at -20°C prior to ELISA analysis.

### ELISA assay

Release of interleukin (IL)-2 and interferon (IFN) γ was assayed on cell culture supernatants (either undiluted or up to 1:5 dilution with RPMI containing 10% FCS as required) using R&D systems ELISA duosets according to manufacturer's instructions.

### Dual-label Immunofluorescence

Tissue blocks were obtained from an area of the lung as far distal to the tumour as possible, and processed as described previously [20]. Lung tissues were cut into 4-μm sections and lifted onto a polysine coated glass slide. For heat induced epitope retrieval in 0.01 M trisodium buffer pH 6 the lung sections were microwaved for 20 min at 800 W. A cocktail of CD8 with either GRα or GRβ primary antibodies diluted in 1.5% normal serum (Vector Labs, Peterborough, UK) with 0.5% triton × 100 was applied overnight at 4°C. Two CD8 primary antibodies were used to avoid applying a cocktail with two antibodies requiring the same species secondary antibody.

According to primary antibody species requirements, CD8 and GR were detected using either Alexa 568 conjugated goat anti-rabbit immunoglobulin IgG (1:200, Invitrogen, Paisley, UK) and Alexa 488 conjugated goat anti-mouse immunoglobulin IgG secondary antibodies (1:200, Invitrogen) respectively. Sections were counterstained with 4,6-diamidino-2-phenylindole (DAPI, Invitrogen). Omission of primary antibody from staining protocol was used as a negative control.

### Image Analysis

The number of CD8^+^GRα^+ ^and CD8^+^GRβ^+ ^cells within inflammatory follicles were calculated and presented as a percentage of the CD8 population. Digital micrographs were obtained using a Nikon Eclipse 80i microscope (Nikon UK Ltd, Surrey, UK) equipped with a QImaging digital camera (Media Cybernetics, Marlow UK) and ImagePro Plus 5.1 software (Media Cybernetics).

### Data analysis & Statistics

IL-2 and IFNγ data were not normally distributed, and so comparisons between groups were performed using a non-parametric ANOVA. If the ANOVA was significant (p < 0.05), then subsequent Mann Whitney U tests were performed for pairwise comparisons. Data for the percentage inhibition by dexamethasone were parametric. Paired t-tests were used to compare the effects of dexamethasone to PHA/PMA alone. Between group comparisons were performed by ANOVA. If the ANOVA was significant (p < 0.05), then subsequent two way comparisons were performed using an unpaired t-test. Immunofluorescence data were normally distributed; comparisons between groups were performed using unpaired t-tests. All analysis was carried out using GraphPad Prism version 5 (GraphPad Software, Inc., San Diego, CA, USA).

## Results

The cell yields/ml of recovered BAL fluid were numerically greater in COPD patients and smokers compared to HNS although this did not reach statistical significance (p = 0.2) (see Table 1). There was no difference between groups in the proportion of lymphocytes present.

### Cytokine Production

**Figure 1:**
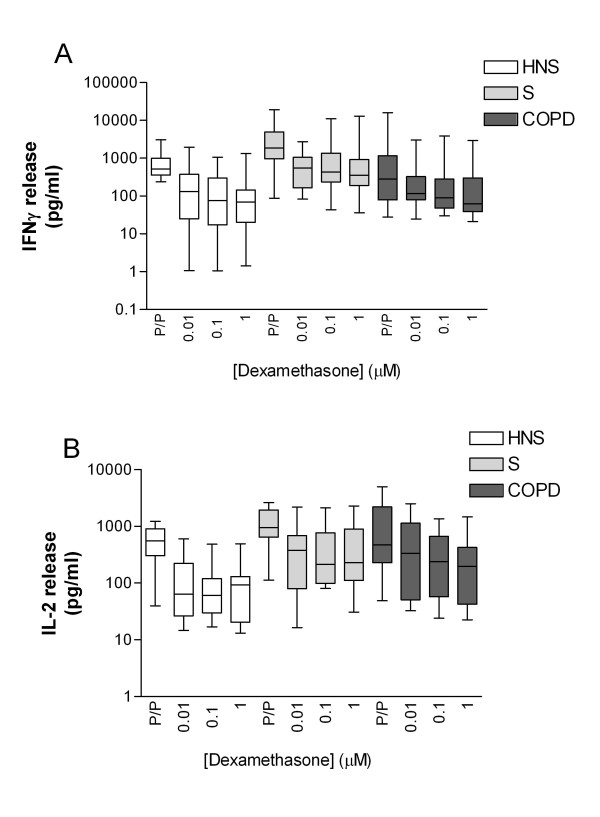
**PHA/PMA induced IFNγ (A) and IL-2 (B) release (pg/ml) from cultured BAL lymphocytes from healthy non-smokers (HNS) (n = 10 light bars), healthy smokers (S) (n = 10 mid grey bars) and COPD patients (n = 14 dark grey bars)**. Cells were pre- treated for 2 hours with dexamethasone (0.01 - 1 μM) prior to 24 hours T cell stimulation with PHA/PMA (P/P) 10 μg/ml each. Data is presented as median (range).

Figure 1 & Table 2 show that unstimulated IFNγ levels and IL-2 levels were not significantly different between subject groups (ANOVA *p *= 0.6 and 0.9 respectively). PHA and PMA stimulation for 24 hours significantly increased the secretion of both IFNγ and IL-2 in all 3 subject groups (*p *< 0.001 for all comparisons of unstimulated vs stimulated levels). There was no statistical difference in stimulated IL-2 levels between groups (ANOVA *p *= 0.2). Stimulated IFNγ levels were different between groups (ANOVA *p *= 0.04), as there were lower levels in HNS compared to smokers (Mann Whitney Unpaired t test *p *= 0.04). There was also significantly lower IFNγ levels in COPD compared to HNS and smokers (Mann Whitney U test *p *= 0.04).

**Table 2:** Basal and stimulated cytokine release from healthy non smokers (HNS), smokers & COPD.

		IFNγ release (pg/ml)	IL-2 release (pg/ml)
**BASAL** **(A)**	HNS	9.8 (3.9-651)	14.5 (6.2-39.8)
	
	Smokers	16.3 (0-75.1)	20.6 (0-453.6)
	
	COPD	17.3 (0-83)	29.6 (0-102.8)
	
	***P *value**	**0.6**	**0.9**

**PHA/PMA** **(B)**	HNS	519 (239.4-3062)	557.6 (39.6-1228)
	
	Smokers	1881 (87.1-19217)	954.9 (112.7-2612)
	
	COPD	284.6 (27.6-15992)	474.2 (28.9-5011)
	
	***P *value**	**0.04**	**0.2**

Data are expressed as median (range).

ANOVAs for basal (A) and stimulated (B) between group comparisons are shown beneath each column

### Dexamethasone effects

**Figure 2:**
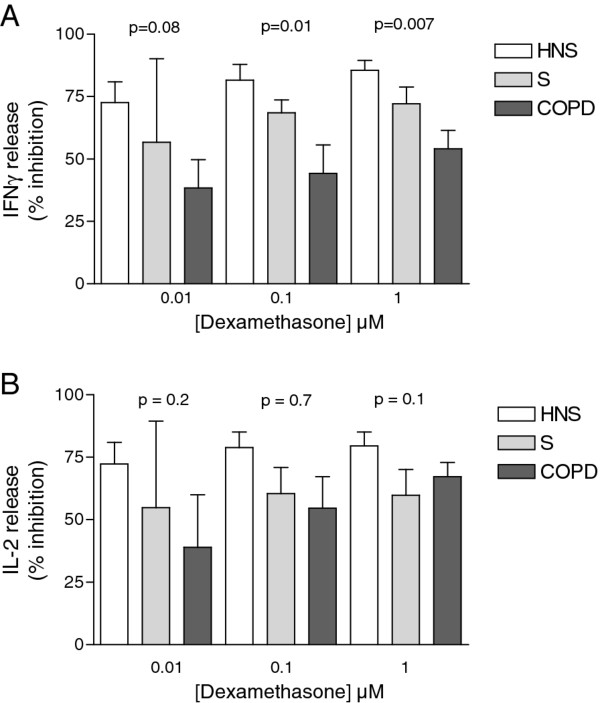
**Reduction of PHA/PMA induced IFNγ (A) and IL-2 (B) release was converted from pg/ml into percent inhibition**. Healthy non-smokers (HNS) (n = 10 light bars), healthy smokers (S) (n = 10 mid grey bars) and COPD patients (n = 14 dark grey bars). Data are expressed as mean (± SD). P-values from ANOVAs for between group comparisons are shown.

Dexamethasone at all concentrations (0.01 - 1 μM) inhibited stimulated IFNγ production in all 3 subject groups; the absolute cytokine levels with and without dexamethasone are shown in Figure 1. As the PHA/PMA stimulated IFNγ levels were different between subject groups, the effect of dexamethasone was normalised to the PHA/PMA levels without dexamethasone and shown in Figure 2 as percentage inhibition. There was a significant difference between groups for the effect of dexamethasone on IFNγ production at 1 μM and 0.1 μM (ANOVA *p *< 0.05 for between group comparisons), with the difference between groups not quite reaching significance at 0.01 μM. The highest concentration of dexamethasone (1 μM) only achieved a mean inhibition of 54.1% of IFNγ production in COPD subjects. Subsequent two way comparisons showed that the inhibition of IFNγ was significantly less in COPD patients compared to HNS at 1 μM (Unpaired t test *p *= 0.001) and 0.1 μM (Unpaired t test *p *= 0.005). At 1 μM there were also significant differences between COPD patients and smokers (Unpaired t test *p *= 0.05), and between HNS and smokers (Unpaired test t *p *= 0.05).

Dexamethasone at all concentrations (0.01-1 μM) inhibited stimulated IL-2 production in all 3 subject groups. There was less inhibition of IL-2 production in COPD patients at all 3 concentrations, but these numerical differences between groups did not reach statistical significance (*p *> 0.05).

Within the COPD group, dexamethasone had similar effects on current and ex smokers for both IL-2 and IFNγ production (*p *> 0.05 for comparisons at each concentration) (data not shown).

### GR expression in follicles

**Figure 3:**
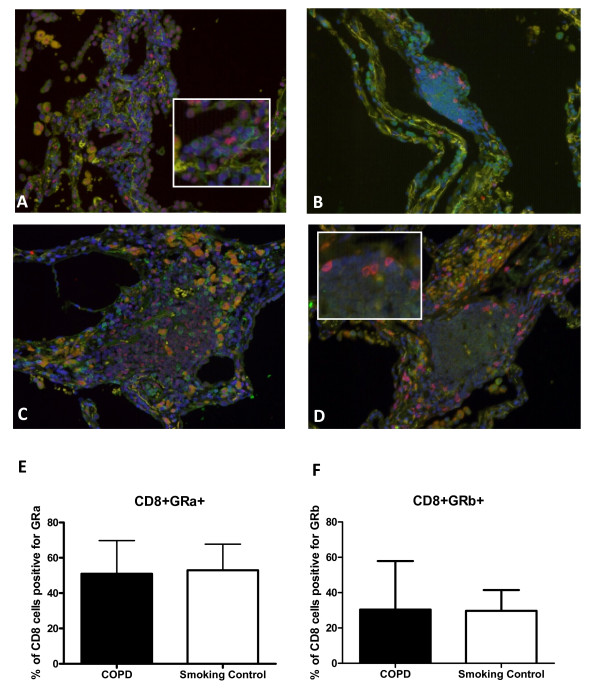
**Representative images for the dual immunofluorescence detection of CD8**^+^**GRα**^+ ^**and CD8**^+^**GRβ**^+ ^**T- cells within human lung tissue from (A&B) a COPD subject and (C&D) a smoking control subject**. (A&C) CD8^+ ^cells were identified using an Alexa-488 conjugated goat anti-rabbit secondary antibody (Green stain) and GRα^+ ^cells were detected using an Alexa-568 conjugated goat anti-mouse secondary antibody (Red stain). (B&D) CD8^+ ^cells were identified using an Alexa-568 conjugated goat anti-mouse secondary antibody (Red stain) and GRβ^+ ^cells were detected using an Alexa-488 conjugated goat anti-rabbit secondary antibody (Green stain). Cell nuclei were counterstained with 4', 6-diamidino-2-phenylindole (DAPI, blue stain). Magnification × 200. (E&F) Analysis of the number of CD8+ cells that were also positive for GRα and GRβ, within COPD (n = 10) and smoking control (n = 11) follicles. Data presented as mean + SD.

There were no significant differences between the percentage of follicular CD8 cells that expressed either GRα or GRβ between COPD patients (means 50.9% and 30.4% respectively) and controls (mean 52.9% and 29.7% respectively), p = 0.8 and 0.9 respectively; see Figure 3 for individual data and representative images.

## Discussion

We show that the sensitivity of airway lymphocytes to corticosteroids is reduced in COPD patients. Our results implicate these cells as important players in the clinical phenomenon of corticosteroid resistance in COPD.

IFNγ plays a key role in Th1 type inflammation and cell mediated cytotoxicity (reviewed [21]). We show that corticosteroids have a reduced inhibitory effect on IFNγ production in COPD patients. The clearest difference in corticosteroid sensitivity observed was between COPD patients and HNS, although there was also evidence of differences between COPD patients and smokers, and between smokers and HNS at the highest dexamethasone concentration (1 μM). These data suggest that smoking itself reduces T lymphocyte corticosteroid sensitivity, and that the development of COPD further reduces corticosteroid sensitivity.

IL-2 is an integral cytokine for T lymphocyte proliferation [22]. There were numerical trends suggesting a reduction in the inhibitory effect of dexamethasone on IL-2 production in COPD patients compared to controls, but these did not reach statistical significance. This indicates that the effects of corticosteroids vary between T lymphocyte cytokines, with IFNγ showing more corticosteroid resistance in COPD patients compared to IL-2. The molecular mechanisms for such a difference have not been explored. The ability of corticosteroids to regulate the activity of transcription factors such as nuclear factor of activated T-lymphocytes (NFAT), activator protein 1 (AP-1), nuclear factor kappa-light-chain-enhancer of activated B cells (NF-κB) and activating transcription factor (ATF) may vary between the promoter regions of the IL-2 and IFNγ genes, and may be further altered by changes such as chromatin modulation that may occur in response to oxidative stress and inflammation in COPD [23].

Corticosteroid insensitivity has been observed in peripheral blood lymphocytes of corticosteroid resistant patients with asthma, ulcerative colitis and rheumatoid arthritis, as well as renal transplant recipients [14-16,24]. IL-2 may play a key role in this phenomenon, as it can induce corticosteroid insensitivity in murine T lymphocytes [25] as well as in PBMCs from healthy volunteers by reducing GR binding affinity [26]. IL-2 mRNA levels are increased in the BAL of steroid resistant patients with asthma [27]. We speculate that IL-2 may play a role in the corticosteroid insensitivity observed in the current study.

IFNγ predisposes the airway microenvironment to a proinflammatory Th1 environment and plays a key role in host defence against viruses [28]. Pulmonary IFNγ levels are increased in COPD patients [29-31], and it has also been reported that COPD severity is related to the degree of IFNγ production by CD8 cells [32]. Viral infections can increase IFNγ levels in COPD patients [33,34].

Our data suggests that that lymphocyte IFNγ production is corticosteroid resistant in COPD patients. This raises the possibility that virally induced IFNγ production in COPD patients is poorly suppressed by corticosteroids; the poor response of viral exacerbations of COPD to corticosteroids is an observation frequently noted by clinicians treating exacerbations.

The number of T lymphocytes in the lungs increases with the severity of COPD [1,35,36]. These T lymphocytes display an increased Th1 response [4,7]. Results in this study suggest that IFNγ could play a central role in persistent Th1 inflammation in COPD, as it responds poorly to corticosteroids. Novel therapies that specifically target IFNγ activity may reduce Th1 inflammation in COPD, such as targeting of the janus kinase (*JAK*)-signal transducer and activator of transcription (STAT) pathway [37].

Our data suggests that IFNγ release from COPD lymphocytes is reduced compared to controls. This finding differs from studies that show increased IFNγ production from COPD lymphocytes [4,7], and increased levels of IFNγ in the airways of COPD patients compared to controls [29-31]. We observed a similar trend for lower IL-2 production in COPD, although this did not reach statistical significance. Reduced cytokine production by alveolar macrophages has been reported from smokers and patients with COPD [38-40] which is thought to be due to a "blunting" effect of cigarette smoke on cell signalling mechanisms such as p38 mitogen activated protein kinase and NFκB [40]. It is possible that a similar effect is also present in COPD lymphocytes. However, as this phenomenon has not been observed in previous studies of COPD lymphocytes [35,36], it is also possible that our findings for both IFNγ and IL-2 are due to random individual variation in a limited sample size rather than a true biological effect.

Corticosteroids bind to GR forming a complex that translocates to the nucleus to interfere with the binding of transcription factors such as NF-κB to the promoter regions of inflammatory genes [11]. A range of molecular mechanisms have been investigated as possible explanations for corticosteroid insensitivity, including the overexpression of the dominant negative splice variant isoform GRβ which is associated with corticosteroid insensitivity in different cell types [41-43] and is reported to be increased in steroid resistant patients with asthma and ulcerative colitis [44-47]. Studies in COPD patients have focused on the role of histone deacetylases which are recruited by ligand activated GR to reverse the unwinding of chromatin that occurs during inflammatory gene transcription; the activity of histone deacetylases has been reported to be reduced in COPD patients, leading to decreased corticosteroid activity [23]. It has been reported that GRα and β expression is reduced in the peripheral lungs of COPD patients compared to controls [48]. However, we observed no difference in either GRα or GRβ expression by CD8 cells within lymphoid follicles in COPD compared to smoking controls. This suggests that altered GR expression level is not the mechanism responsible for the corticosteroid insensitivity observed in the current study. We did not have access to sufficient samples from non-smoking controls for this analysis, as the majority of lung cancer patients are current or ex-smokers. Corticosteroid insensitivity is probably both a cell type and disease specific phenomenon, and further studies should address the mechanisms responsible for the corticosteroid resistance in COPD airway lymphocytes observed in the current study.

## Conclusions

In conclusion, we have shown that T lymphocytes from the airways COPD patients display corticosteroid insensitivity. The mechanisms responsible for our findings require further study. Airway lymphocytes may play a key role in the corticosteroid insensitivity observed in COPD patients.

## Abbreviations

AP-1: Activator protein 1; ATF: Activating transcription factor; BAL: Bronchoalveolar lavage; COPD: Chronic Obstructive Pulmonary Disease; DAPI: 4,6-diamidino-2-phenylindole; FCS: Fetal calf serum; FEV_1: _Forced expired volume in 1 second; FVC: Forced vital capacity; GR: Glucocorticoid receptor; HNS: Healthy non smoker; IFN: Interferon; IL: Interleukin; NFAT: Nuclear factor of activated T-cells; NF-κB; Nuclear factor kappa-light-chain-enhancer of activated B cells; PHA: Phytohaemagglutinin; PMA: Phorbol myristate acetate; S: Smokers.

## Competing interests

Manminder Kaur, Lucy Smyth, Paul Cadden, Seamus Grundy and Jonathan Plumb, have no competing interest to declare. Dave Singh and David Ray are supported by research grants as outlined in their personal declarations.

## Authors' contributions

MK: carried out main body of lab work, data analysis and manuscript composition. LS: carried out main body of lab work and manuscript composition. PC: involved in subject recruitment, research bronchscopies, contribution to lab work and data analysis. SG: subject recruitment, research bronchsocopies and data analysis. JP: carried out the immunofluoresence work and contributed to the manuscript. DR: involved in study design, data analysis and manuscript composition, DS: involved in study design, data analysis and senior contribution to manuscript composition.
